# Prevalence of dry eye syndrome in association with the use of contact lenses in Saudi Arabia

**DOI:** 10.1186/s12886-021-01912-8

**Published:** 2021-03-23

**Authors:** Abeer Habeeb Almutairi, Bayan Sulaiman Alalawi, Ghadir Hamzah Badr, Razan Ahmed Alawaz, Maan Albarry, Hossein M. Elbadawy

**Affiliations:** 1 College of Medicine, Taibah University, Madinah, Kingdom of Saudi Arabia; 2 Department of Ophthalmology, College of Medicine, Taibah University, Madinah, Kingdom of Saudi Arabia; 3 Department of Pharmacology and Toxicology, College of Pharmacy, Taibah university, Madinah, Kingdom of Saudi Arabia

**Keywords:** Dry eye syndrome, Saudi Arabia, Contact lenses, Prevalence, Survey

## Abstract

**Background:**

Dry eye disease is a tear film disorder which can cause discomfort to patients and negatively affect vision acuity. A number of risk factors has been reported to affect the incidence and severity of dry eye syndrome (DES). The aim is to study the prevalence of DES in Saudi Arabia and the factors affecting the severity of DES in relation to the use of contact lenses.

**Methods:**

A cross-sectional questionnaire-based study was conducted on 310 participants using the ocular surface disease index (OSDI) questionnaire and the eye dryness part from contact lens questionnaire-8 (CLDEQ-8). Dry eye OSDI scores were compared across different epidemiological and risk factors with focus on the use of contact lenses. Pearson and Spearman’s correlation coefficients were used to analyze the frequency of contact lenses usage in relation to OSDI scores. Student’s t-test and one-way analysis of variance (ANOVA) tests were used to compare means of two or more than two groups, respectively.

**Results:**

Forty eight (15.5%) of participants did not have any degree of DES, achieving an OSDI score between 0 and 12. Forty participants (12.9%) scored from 13 to 22, (mild DES), 44 (14.2%) were moderate, scoring 23–32 on the OSDI, while those who scored above 33 were 178 (57.4%) had severe DES. The mean score for all participants was 37.8. A high percentage of participants (84.5%) had some degree of DES. There was a strong positive correlation between OSDI score and the frequency of the feeling of dry eye and a moderate positive correlation between OSDI score and the intensity of dryness feeling. Out of 310 participants, 136 (43.9%) indicated using contact lenses. There was no significant association between the use of contact lenses per se and DES, however, those who used contact lenses more frequently had significantly higher OSDI scores.

**Conclusions:**

Dry eye syndrome is a widespread, underdiagnosed condition in Saudi Arabia. The frequency of contact lenses use may contribute to the incidence of DES.

**Supplementary Information:**

The online version contains supplementary material available at 10.1186/s12886-021-01912-8.

## Background

Dry eye syndrome (DES), also known as keratoconjunctivitis sicca, is a chronic disease with several underlaying pathologies. It is a tear film disorder occurring due to increase tear evaporation or deficiency of tear production which causes interpalpebral ocular surface damage associated with ocular discomfort symptoms [1]. These symptoms include eye dryness, foreign body sensation, and burning sensation result in eye redness and discomfort. At more advanced stages, blurred vision may occur with eye discharges which gradually worsen the vision acuity on the long term. The condition is aggravated usually at hot, dry climate which is specifically relevant to Saudi Arabia [2–6]. The prevalence of DES can range between 7 and 34% with variations dues to the type of diagnosis or the population [7, 8]. Deficiency in the tear film is the main cause of the disease. This occurs secondary to the high evaporation rate or insufficient tear production. When the lacrimal gland function is adversely affected, it consequently decreases tear volume, leading to aqueous deficiency [9, 10].

About 14% of people above the age of 65 report symptoms of dry eye [11]. Beside age, several causes were reported as risk factors of DES as being a female, smoking, or undergoing a laser in situ keratomileusis operation [12, 13]. Saudi population is at risk of DES due to several environmental and epidemiological risk factors [14] along with the increasing rate of using contact lenses especially among female medical students [15]. As in most of Saudi Arabia regions, hot desert climate with a temperature reaching up to 50 °C in the summer is predominant in many regions of the country. Previous studies have focused on the prevalence of DES and risk factors related to the incidence of DES, reporting how DES may affect the quality of life. A limited number of studies, however, investigated the possible relationship between using contact lenses and the incidence and severity of DES. Since DES is considered as one of the most common complaints in ophthalmology field [14], this study aimed at assessing the prevalence of DES in Saudi Arabia in different regions throughout the kingdom. Additionally, the relationship between the severity of DES and various other factors which may contribute to the incidence and the severity of the disease were investigated.

## Methods

### Study design and settings

This is an exploratory cross-sectional questionnaire-based study conducted among Saudi Arabia population aged from 15 years old and above, living in the Kingdom of Saudi Arabia from the 10th of August until the 15th October 2020. Informed consents were obtained from all the participants after describing the aim of the study. For minors below 18 years of age, an informed consent was obtained from legally authorized representatives. The Privacy and confidentiality of patients’ information are anonymous and preserved. Both quantitative and qualitative questions were used in this study. The questionnaire evaluated the prevalence of dry eye syndrome among Saudi Arabia population and its relationship with contact lenses use. Previous history of eye surgeries, ocular infections, use of drugs which affect the tear film, ocular defects and individuals less than 15 years of age were the exclusions criteria considered. All participants were not diagnosed before with DES.

### The ocular surface disease index (OSDI)

﻿The OSDI questionnaire [16] on dry eye symptoms and the eye dryness part from Contact lens questionnaire-8 (CLDEQ-8) [17] were translated into Arabic and used to interview the subjects in different regions within the Kingdom of Saudi Arabia. The questions elucidated the ocular symptoms of all participants which occurred during a week prior to filling up the questionnaire. The full questionnaire, attached as a supplementary file to this article, started with social and demographic data of each participant and whether they currently use contact lenses or not. The questionnaire was divided into three subdivisions. Those are general eye complaints, vision quality and causative agents and factors. The grading used was on a scale from 0 to 4, where 0 denoted none; 1, some; 2, half; 3, most; and 4, all of the time. The total score is commonly estimated on the basis of the following formula; OSDI = [sum of the scores for all questions answered × 100]/total number of questions answered] × 4 [16]. The contribution of contact lenses to the pathology, incidence and severity of the DES was studied to determine the frequency of these events. The total score was calculated on a scale of 0 to100, with higher scores representing greater distress. Scores 0 to 12 representing normal, 13 to 22 representing mild dry eye disease, 23 to 32 representing moderate dry eye disease, and greater than 33 representing severe dry eye disease. Statistical analysis was performed utilizing the Statistical Package for Social Sciences (SPSS). Categorical variables were described in the form of frequency and percentage whereas continuous variables were described in the form of mean and standard deviation [18]. Pearson and Spearman’s correlation coefficients were used to compare were used to analyze the frequency of contact lenses usage. Student’s t-test and one-way analysis of variance (ANOVA) tests were used to compare means of two or more than two groups, respectively. Statistical significance was determined at *p* ≤ 0.05. All data generated and analyzed during this study are included in this published article and its supplementary information files. The protocol for research involving humans was in accordance to guidelines of national research ethics regulations and according to the Declaration of Helsinki. Ethical approval was obtained from Taibah University Research Ethics Committee number TUCDREC/230720/HMelbadawy.

## Results

### Characteristics of the participants

All regions of Saudi Arabia were covered with 46.5% of participants from the western region, where the study team is based. Questionnaires were distributed electronically via an online platform. Most of participants were university graduates living in cities. The number of contact lens users was 136 (43.9%), while 174 (56.1%) were non-contact lens users. Frequency of using contact lenses was also examined; the participants were asked how often they use contact lenses. Thirty-four participants indicated that they use contact lenses every day, 50 participants used it on weekly basis, 37 monthly and 15 participants answered that they use contact lenses only once a year. (Table 1).

**Table 1:** Descritive data on the charectersitics of study population

		Frequency	Percent
Sex	Female	252	81.3
	Male	58	18.7
Nationality	Saudi	293	94.5
	Non-Saudi	17	5.5
Region	Western	144	46.5
	Middle	108	34.8
	Eastern	26	8.4
	Northern	16	5.2
	Southern	16	5.2
Age	Less than 18	37	11.9
	18–24	179	57.7
	25–30	60	19.4
	31–40	23	7.4
	41–50	9	2.9
	More than 50	2	0.6
Marital status	Single	247	79.7
	Married	60	19.4
	Separated	2	0.6
	Widowed	1	0.3
Employment	Unemployed	235	75.8
	Employee	73	23.5
	Retired	2	0.6
Education	Uneducated	2	0.6
	Read and write	1	0.3
	Intermediate school	5	1.6
	High school	79	25.5
	University	223	71.9
Residency	Rural	29	9.4
	Urban	281	90.6
Monthly income	Less than 5000	162	52.3
	5000 to 10,000	68	21.9
	More than 10,000	80	25.8
Smoking	Non smoker	280	90.3
	Smoker	30	9.7
Usage of contact lenses	No	174	56.1
	Yes	136	43.9
Frequency of usage	Yearly	15	11.0
	Every 6 months	37	27.2
	Monthly	50	36.8
	Daily	34	25.0

### Eye dryness sensation as an indicator for DES

The intensity of dry eye sensation was evaluated on a scale of 1 to 5, where 1 indicated that the feeling of dryness was not at all intense, while 5 indicated a very intense feeling of eye dryness. A low percentage of participants (9.6%) reported that they did not have any feeling of eye dryness, while 15.4% indicated that the dryness feeling was not at all intense. On the scale of 5, 27.2% scored 2, 21.3% scored 3 and 147% scored 4. Very intense feeling of eye dryness was reported by 11% of the study population. Regarding the frequency of dry eye sensation, 17.1% did not report any dry eye sensation during the 2 weeks before filling up the questionnaire, while 19.5% rarely felt some dry eye sensation. On the other hand, 28.8% occasionally complained from dry eye sensation and 22.2% experienced this feeling more frequently. However, 12.5% of participants had this feeling constantly (Table 2) .

**Table 2:** Intensity of dry eye sensation

		Frequency	Percent
When your eyes felt dry, how intense was this feeling of dryness at the end of your wearing time?	Never had it	13	9.6
	1- Not at all intense	21	15.4
	2	37	27.2
	3	29	21.3
	4	20	14.7
	5- Very intense	15	11.0
During a typical day in the past 2 weeks, how often did your eyes feel dry?	Never	44	17.1
	Rarely	50	19.5
	Sometimes	74	28.8
	Frequently	57	22.2
	Constantly	32	12.5

### The distribution of OSDI scores

**Fig. 1:**
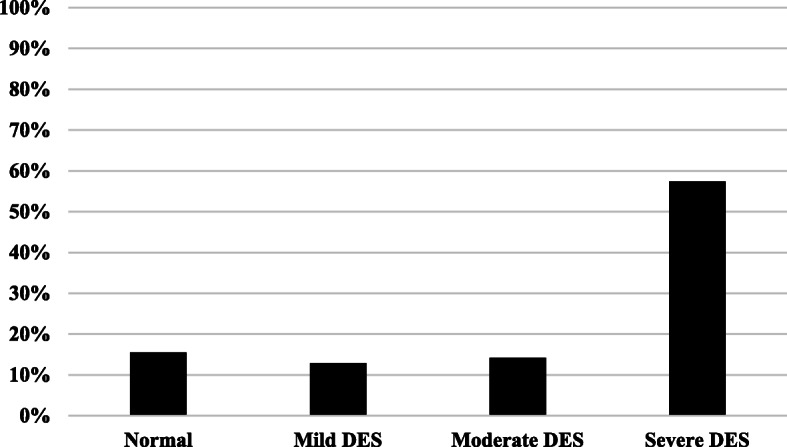
OSDI score distribution according to severity. The figure illustrates the distribution of OSDI score according to overall score. OSDI scores were distributed as normal 0–12, mild DES 13–22, moderate DES 23–32 and severe DES above 33. Frequency is the number of participants scoring OSDI within each designated score

The OSDI score ranged from 0 to 100 showing all range of DES, but the mean score was 37.8, which is well above the threshold (OSDI 20) (Table 3). The distribution of OSDI scores among participants (Fig. 1) shows that the majority of participants scored below 50 OSDI score, with a reasonable, yet alarming, percentage of the scores above 50.

**Table 3:** The OSDI overall score

	N	Minimum	Maximum	Mean	SED
OSDI score	310	0.0	100.0	37.8	21.2

### Prevalence of DES in Saudi Arabia

**Fig. 2:**
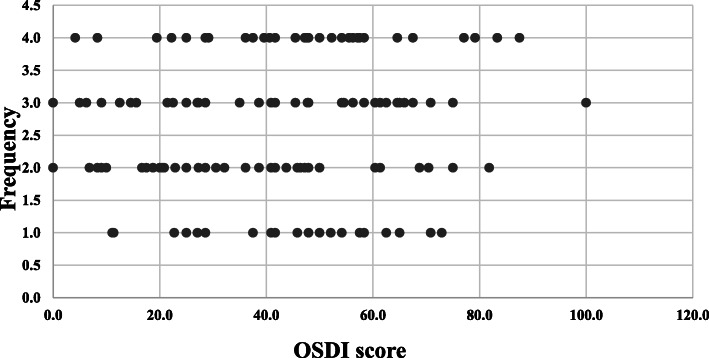
The OSDI scores of all participants. A scatter plot showing the breakdown of OSDI scores distributed from all participants. The X axis shows the OSDI score and the Y axis shows the frequency of each score

Using the OSDI questionnaire, participants were classified according to the severity of DES into four groups; those who scored 0–12 were normal subjects, 13–22 had mild DES, 23–32 were considered having moderate DES while a score above 33 was considered as severe DES. It was found that 84.5% of the subjects who participated in this study had some degree of DES complaints. However, 57.4% had severe DES according to the OSDI score (Fig. 1). An OSDI score distribution of all participants us shown in Fig. 2, indicating the frequency for each score.

### Different factors affecting the incidence of DES

Respondents to the questionnaire were grouped into different groups denoting factors which may affect the incidence and severity of DES. Comparing the OSDI scores of those groups, it was found that contact lenses use was not correlated to higher OSDI score. Education level, employment status, income and residence area were not correlated to OSDI scores. Although smokers and those living in urban rather than rural areas of Saudi Arabia seemed to have slightly higher OSDI scores (Table 4).

**Table 4:** Risk factors and socioepidemiological factors related to OSDI scores

		N	Mean OSDI	SD
Using contact lenses	No	174	36.1	21.4
	Yes	136	39.9	20.8
Sex	Male	58	34.3	22.7
	Female	252	38.6	20.8
Nationality	Saudi	293	37.5	20.8
	Non-Saudi	17	43.0	26.1
Marital status	Married	60	39.6	23.3
	Not married	250	37.4	20.6
Employment	Employed	73	36.1	21.1
	Not employed	237	38.3	21.2
Education	University degree	223	37.0	20.9
	Less than university degree	87	39.7	21.7
Residence	Rural	29	35.7	20.7
	Urban	281	38.0	21.2
Smoking	Non smoker	280	37.3	20.6
	Smoker	30	42.1	26.0
Income	Less than 5000 SR	162	38.6	21.4
	5000 to 10,000 SR	68	38.3	22.1
	more than 10,000 SR	80	35.8	20.0

SR = Saudi Riyal

### Correlation between the contact lens usage and OSDI

Chi square test used to study the association between using contact lenses and different levels of eye dryness (Table 5), there was no statistically significant association between those two variables (*p*-value = 0.422). Analysis was performed using Pearson correlation coefficient and Spearman’s correlation coefficient to study the relation of OSDI score and the frequency of wearing contact lenses. Using contact lenses more frequently was associated with high OSDI score. Moreover, the frequency and intensity of dry eye sensation was positively correlated to the frequency of using contact lenses (Table 6).

**Table 5:** Statistical analysis of the relation between the use of contact lenses and the incidence of DES

Dry eye		Using Contact lenses		*P* value
		No	Yes	
Normal	N	32	16	0.422
	%	18.4%	11.8%	
Mild dry eye	N	23	17	
	%	13.2%	12.5%	
Moderate dry eye	N	23	21	
	%	13.2%	15.4%	
severe dry eye	N	96	82	
	%	55.2%	60.3%

**Table 6:** Statistical analysis on the frequency and intensity of DES in relation to the use of contact lenses

	OSDI	
AGE	Pearson Correlation Coefficient	−0.057
	P value	0.320
During a typical day in the past 2 weeks, how often did your eyes feel dry?	Spearman’s Correlation Coefficient	0.440
	P value	< 0.001
When your eyes felt dry, how intense was this feeling of dryness after at the end of your wearing time	Spearman’s Correlation Coefficient	0.227
	P value	0.008
Frequency of contact lens usage	Spearman’s Correlation Coefficient	0.179
	P value	0.037

## Discussion

A cross suctional study was conducted in Saudi Arabia using the (OSDI) questionnaire previously established and validated by the Outcomes Research Group (Allergan, USA). The OSDI questionnaire, comprising twelve questions, is intended to assess the symptoms of ocular irritation and DES. A higher OSDI score is associated with a higher incidence of dry eye. Dry eye syndrome is thought to be widespread in Saudi Arabia [19] due to the dry and hot weather most times of the year. In a study performed in the eastern region of Saudi Arabia, it was found that the prevalence of DES was significantly associated with female gender, old age and history of diabetes [20]. However, our findings were contrary. We did not find correlations between gender and the incidence of DES, which comes in agreement with another study carried out in the western region of Saudi Arabia in 2009 [19]. Some previous studies found that almost all Saudi population may have some degree of DES [19, 21], with one study showing that prolonged screen time can be a main contributor to the incidence of DES [21]. In our study, the prevalence of DES was over 85%. We classified the patients according to severity and we found that more than half of our sample (57.4%) were having severe DES. According to the data from this work and previously reported data, the prevalence of DES is believed to vary depending on how the disease is diagnosed and which population is surveyed. For example, the prevalence of DES in Australia was estimated to be 10.8% only [22]. The study should have used a recruitment strategy to ensure better distribution of the sample. Most of the study participants were young (57.7% aged 18–24 years old) females (81.3%), which does not reflect the normal distribution of the population in Saudi Arabia. Additionally, the study did not cover all areas of Saudi Arabia, because of limitations in the ability to distribute questionnaires in these areas. This great variation can be attributed to differences in environmental, genetic and lifestyle factors. Importantly, the clinical diagnosis by tear film breakup tests is more accurate than using questionnaires and DES scoring systems but it more costly, and the method will affect the sample size, which is a limitation of this study. However, the OSDI, used in this study shows a reliable tool for identifying DES [16]. Regarding the use of contact lenses, per se, it was not associated with DES, although it has been reported previously that wearing contact lenses cause or worsen DES [17]. However, a significant correlation was found between the OSDI score and the frequency of contact lenses use. Therefore, it was concluded that using contact lenses more frequently is a main cause of high OSDI score.

## Conclusions

This work highlighted the fact that DES is prevalent in Saudi Arabian population above internationally reported rates, and requiring further attention to the causes of the disease and the management of the underlying pathology. Wearing contact lenses, per se, was not a correlated with OSDI score, while using contact lenses more frequently was significantly correlated to high OSDI score. Contact lenses, therefore, can be a contributing factor along with other factors, but not the main nor the only cause.

## Supplementary Information

**Additional file 1.**

**Additional file 2.**

## Data Availability

All data generated or analyzed during this study are included in this published article.
