# Supplementary material for: Prevalence of dry eye syndrome in association with the use of contact lenses in Saudi Arabia
Source: BMC Ophthalmol. 2021 Mar 23;21:147. doi: 10.1186/s12886-021-01912-8 (PMC7986502; doi:10.1186/s12886-021-01912-8)
Supplement: Supplementary file 1 — Additional file 1. [file 12886_2021_1912_MOESM1_ESM.docx]

**Prevalence of Dry Eye Syndrome in association with the use of contact lenses in Saudi Arabia**

Abeer Habeeb Almutairi^1^, Bayan Sulaiman Alalawi^1^, Ghadir Hamzah Badr^1^, Razan Ahmed Alawaz^1^, Maan Albarry^2^, Hossein Mostafa Elbadawy^3^

Affiliations:

1 College of Medicine, Taibah University, Madinah, Kingdom of Saudi Arabia

2 Department of Ophthalmology, College of Medicine, Taibah University, Madinah, Kingdom of Saudi Arabia. Zip 42353

3 Department of Pharmacology and Toxicology, College of Pharmacy, Taibah university, Madinah, Kingdom of Saudi Arabia

Corresponding author:

Abeer Habeeb Almutairi, College of Medicine, Taibah University, Universities road, Madinah, Kingdom of Saudi Arabia.

Email: Abeerhabeeb77@gmail.com

Phone: 00966504322751

**The questionnaire**

Informed Consent:

You are being asked to voluntarily participate in this survey research study. The purpose of the study is to know the Prevalence of Dry Eye Syndrome in association with use of contact lenses in Saudi Arabia. The work also aims at finding any possible relationships between Dry eye syndrome and the use of contact lenses.

If you agree to participate, your participation will involve completing a survey. It should take no more than 10 minutes. Your name will not appear on your completed survey, and no identifying information is being collected as part of this survey.

Any questions you have will be answered. There are no known risks from your participation. No direct benefit from your participation is expected. There is no cost to you except for your time. You will not be paid for participation in this study.

Only the study team will have access to the information that you provide, which will remain anonymous.

You can obtain more information from the Principal Investigator:

Dr. Abeer Almutairi (medical intern)

Mobile: 966504322751

E-mail: abeer-almemony@hotmail.com

Department: college of medicine- Taibah university.

Completing this survey indicates your voluntary agreement to participate. By participating in the survey, you are giving permission for the investigator to use your information for research purposes.

Thank you.

Consent Agreement:

- I agree to participate.
- I disagree to participate.

**Personal data:**

**Gender:**

- Male
- Female

Nationality:

- SAUDI
- NON-SAUDI

Region: (regions of Saudi Arabia)

- Middle region
- West region
- East region
- North region
- South region

Age:

- Less than 18
- 18-24
- 25-30
- 31-40
- 41-50
- More than 50

Marital status:

- Single
- married
- Divorced
- Widowed

Occupation:

- Employed
- unemployed
- Retiree

Educational level:

- Illiterate
- Reed/ and write
- Primary school
- Intermediate school
- Secondary school
- University.

Residence:

- Urban
- Rural.

Month Salary:

- Less than 5000
- 5000-10000
- More than 10000

Are you smoker:

- Yes
- No

**Contact lens use:**

Are you using contact lenses?

- Yes
- No

How many times you are wearing contact lenses?

- Daily.
- Monthly.
- Every 6 months.
- Yearly.

CONTACT LENS QUESTIONNAIRE-8 (CLDEQ-8):

Questions about EYE DRYNESS:

a. During a typical day in the past 2 weeks, how often did your eyes feel dry?

- 0  Never
- 1  Rarely
- 2  Sometimes
- 3  Frequently
- 4  Constantly

When your eyes felt dry, how intense was this feeling of dryness...

b. At the end of your wearing time?


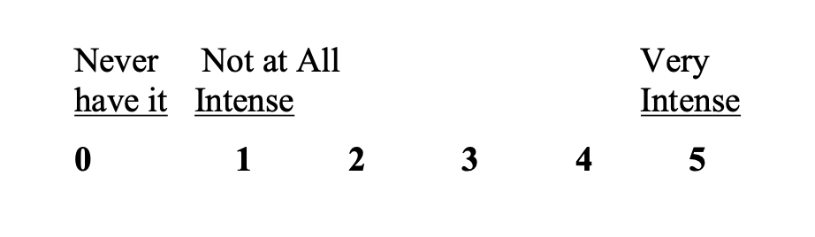


Thank you.
